# Linear and Non-Linear Optical Imaging of Cancer Cells with Silicon Nanoparticles

**DOI:** 10.3390/ijms17091536

**Published:** 2016-09-12

**Authors:** Elen Tolstik, Liubov A. Osminkina, Denis Akimov, Maksim B. Gongalsky, Andrew A. Kudryavtsev, Victor Yu. Timoshenko, Rainer Heintzmann, Vladimir Sivakov, Jürgen Popp

**Affiliations:** 1 Leibniz Institute of Photonic Technology, Jena 07745, Germany; denis.akimov@ipht-jena.de (D.A.); heintzmann@gmail.com (R.H.); vladimir.sivakov@ipht-jena.de (V.S.); juergen.popp@ipht-jena.de (J.P.); 2 Physics Department, Lomonosov Moscow State University, Moscow 119991, Russia; osminkina@mig.phys.msu.ru (L.A.O.); mgongalsky@gmail.com (M.B.G.); timoshen@physics.msu.ru (V.Y.T.); 3 Interational Laboratory “Bio-Nanophotonics”, National Research Nuclear University “Moscow Engineering Physics Institute”, Moscow 115409, Russia; 4 Institute of Theoretical and Experimental Biophysics, Russian Academy of Science, Pushino 142290, Russia; centavr42@mail.ru; 5 Institute of Physical Chemistry, Abbe Center of Photonics, Friedrich-Schiller-University, Jena 07743, Germany

**Keywords:** silicon nanoparticles, nanowires, multimodal bioimaging, high-resolution structured illumination microscopy (HR-SIM), Raman spectroscopy, coherent anti-Stokes Raman scattering (CARS), two-photon excited fluorescence (TPEF)

## Abstract

New approaches for visualisation of silicon nanoparticles (SiNPs) in cancer cells are realised by means of the linear and nonlinear optics in vitro. Aqueous colloidal solutions of SiNPs with sizes of about 10–40 nm obtained by ultrasound grinding of silicon nanowires were introduced into breast cancer cells (MCF-7 cell line). Further, the time-varying nanoparticles enclosed in cell structures were visualised by high-resolution structured illumination microscopy (HR-SIM) and micro-Raman spectroscopy. Additionally, the nonlinear optical methods of two-photon excited fluorescence (TPEF) and coherent anti-Stokes Raman scattering (CARS) with infrared laser excitation were applied to study the localisation of SiNPs in cells. Advantages of the nonlinear methods, such as rapid imaging, which prevents cells from overheating and larger penetration depth compared to the single-photon excited HR-SIM, are discussed. The obtained results reveal new perspectives of the multimodal visualisation and precise detection of the uptake of biodegradable non-toxic SiNPs by cancer cells and they are discussed in view of future applications for the optical diagnostics of cancer tumours.

## 1. Introduction

Recently, silicon nanoparticles (SiNPs) are widely studied as promising biocompatible and biodegradable agents for medical purposes [1,2,3]. This high interest is motivated by unique advantages of SiNPs as multimodal labels for cancer diagnostics both in vitro and in vivo [1,3,4,5] and as nano-containers for drug delivery [3,6,7,8,9,10]. The enhanced accumulation of SiNPs in tumours enables an accurate detection and distinction of tumour’s boundaries. Moreover, Si-based nanomaterials can be used as active agents for cancer therapy due to their sensitizing properties under photo- [10,11,12,13], sono- [13,14] and electromagnetic radio-frequency [15] stimulus. 

SiNPs for biomedical purposes can be prepared by various methods such as mechanical milling of porous silicon (PSi) films [12,13,14], ultrasonic fragmentation of PSi [8] or silicon-nanowires (SiNWs) [16], laser ablation of crystalline silicon (c-Si) targets [12], microwave plasma synthesis [13], etc. Nanocrystals of silicon with sizes of 2–5 nm are known to exhibit room temperature photoluminescence (PL) in the visible and near-infrared (NIR) spectral regions [14]. The PL properties of SiNPs have been successfully used for bioimaging [8,12,17] and biosensing [8,10,15,16]. Recently, a method of high-resolution structured illumination microscopy (HR-SIM) has been applied to monitor the uptake and intracellular distribution of porous SiNPs in cancer cells [17]. However, the HR-SIM visualisation of SiNPs requires typically efficient photoexcitation with UV or blue light sources, which can induce undesirable light scattering, obstructing autofluorescence of cells and even their photo-damage. Moreover, non-luminescent SiNPs are very promising for non-optical therapeutic modalities [9,13,14,15]. Therefore, a search of alternative tools to monitor SiNPs during bio-visualisation and cancer diagnostics is of great importance.

Micro-Raman spectroscopy or hyperspectral Raman imaging, a microscopic imaging technique combining a microscope with a Raman spectrometer, has the advantage to identify subcellular structures by exciting certain molecular vibrations of the chemical composition of complex biological objects [18,19,20]. Therefore, the hyperspectral Raman imaging enables an accurate diagnosis and differentiation between malignant and benign cells with sub-micrometre resolution due to chemical differences of them [20]. Additionally, the Raman spectroscopy plays a significant role in drug delivery monitoring in vitro and in vivo [19]. This non-invasive label-free technique allows realizing the time-resolved 3D mapping of the distribution of active pharmaceutical ingredients and nanoparticle drug containers and to visualise their intracellular penetration and subsequent drug release [18,19,21,22,23]. Besides, Raman spectroscopy was successfully applied as a diagnostic tool for breast, skin, prostate, colon and liver cancer [24,25,26]. In this context, Raman microscopy is a promising tool to study biodegradable SiNPs inside tumour cells and it can be used for cancer theranostics [17]. The micro-Raman spectroscopy in combination with fluorescence measurements possessed a high level of efficiency for investigations of toxicity, stability and intracellular uptake of various metal-oxide nanomaterials, e.g., TiO_2_ [27], ZrO_2_ [28], as well as carbon dots [29]. Nonetheless, the hyperspectral Raman imaging technique belongs to relatively slow diagnostic methods, concerning its potential application for the real-time diagnostics of cancer.

Multifunctional nanoparticles (NPs) are known to be promising for the visualisation and therapy of cancer in vitro and in vivo [30]. For examples, fluorescent carbon dots [29,31], polymer/quantum dots nanocomposites [32], fluorescent dye-doped silica NPs, quantum dots, gold NPs [33], polysaccharide analogues conjugated with a fluorescent dye [34] etc., possess the cancer diagnostics by means of linear optical methods. The size, shape and electrical charge of NPs are important for the cellular uptake, i.e., charged NPs can be taken up more rapidly by cells than neutral ones [35]. Multimodal visualisation of cells can be realised by using NPs, which combine both the fluorescent and magnetic properties [36]. While the combination of magnetic and optical imaging can provide complementary information about the target objects, many conventional magnetic contrast agents, e.g., gadolinium based ones, are rather toxic and they require a strong conjugation with neutral organic compounds [36].

Recently, coherent anti-Stokes Raman scattering (CARS) and two-photon excited fluorescence (TPEF) microscopy were applied for investigation of biosystems [37,38]. The CARS intensity, similar to the Raman spectroscopy, is resonant with the vibration frequencies of chemical bonds. However, opposite to imaging based on spontaneous Raman scattering, a CARS image can be recorded in a single shot, opening up the possibility of 3D imaging of fast dynamical phenomena at high speed, which is limited only by the laser-pulse repetition rate. Therefore, the CARS microscopy is about five orders of magnitude faster than the micro-Raman spectroscopy. Unlike the Raman spectroscopy, the CARS provides a coherent signal, which is orders of magnitude stronger than the spontaneous Raman emission, thus ensuring a much higher sensitivity. The four-wave mixing process, applied by CARS and involving pump and Stokes beams interactions with the sample, generates a resonantly enhanced CARS signal at the anti-Stokes frequency, allowing high resolution and thus selective imaging on chemical and molecular levels [37,38,39]. In other words, whereas the spontaneous Raman scattering is an incoherent superposition of the signals from individual molecules and, therefore, is linear in the concentration of these molecules, the CARS signal is generated by a coherent addition of signals and increases super-linearly with molecule number and pump intensity. Note, an undesirable non-resonant background, which limits the contrast and spectral selectivity of CARS images, has to be suppressed additionally [38]. 

TPEF imaging is typically based on two-photon absorption followed by emission of the fluorescence photon [40]. An improvement of spatial resolution, especially in axial direction, is caused by reducing the excitation volume, because of the tight focal spot with consequently higher photon density, thus decreasing undesirable bleaching of the sample and out-of-focus fluorescence [41]. While the one-photon excitation can be done with a simple continuous-wave source of light, the CARS and TPEF techniques require a high flux of the excitation photons, which is usually generated by a NIR femtosecond laser. The use of NIR excitation can also minimise the light scattering in tissue. Due to the nonlinear origin of the absorption the background of TPEF is strongly suppressed, which leads to an increased depth of the visualisation with reduced scattering artefacts and higher sensitivity and specificity of analysed species and bonds [41,42,43,44]. The ability of three-dimensional sectioning of CARS and TPEF microscopies has been used to perform non-invasive optical biopsy of tissues [45] and to visualise thick tissue samples transfected with different NPs and drug delivery systems such as metal oxide NPs, gold nanorods and nanoshells as well as carbon nanotubes [34,43,44,45,46,47,48,49,50,51,52]. These non-linear optical approaches reveal a high ability to monitor polymer [53] and gold [54] NPs in living systems. Among promising TPEF materials for non-linear optical imaging, CdSe quantum dots and related core-shell NPs [55], carbon dots [56], silicon carbide NPs [57], zinc oxide nanocrystals [58], gold nanorods [59], etc. were proposed. Despite the tremendous progress achieved by using quantum dots or organic molecules for bioimaging, some problems remain to be solved: (i) cyto- and genotoxicity; (ii) slow clearance; (iii) accumulation in the organism; and (iv) low chemical stability. In this view, biodegradable and non-toxic Si-based nanomaterials seems to be good alternatives.

The TPEF technique was used to visualise Mn-doped Si quantum dots in cells in vitro [60]. Circulation of SiNWs in blood vessels and their final distribution in tissue were investigated by non-linear optical methods of four-wave mixing and third harmonic generation [61]. While a strong CARS signal was observed for SiNW arrays [62], there is no information on applications of this approach for bioimaging.

Thus, a combination of the linear techniques of HR-SIM and hyperspectral Raman imaging with the non-linear methods of CARS and TPEF provides possibilities to investigate biosystems and to identify accurately different cancer regions [37,41,45,63]. These spectroscopic imaging techniques were successfully applied for the investigation of materials used in medical sciences and, as well as, for studies of living organisms [40,42,44,64]. With these methods, it is possible to perform label-free characterisation of human skin [25,64,65,66], colon tissue [42], lipid metabolism, and DNA and RNA distributions through cell cycle [63], atherosclerosis [37] and proteins [38]. By applying both linear and non-linear techniques, a significant breakthrough in cancer research and diagnostics has recently been demonstrated [26,41,45,63,64,67,68]. On the other hand, Si-based nanomaterials with strong vibrational resonances in combination with the spectroscopic approaches mentioned above allow avoiding completely the fluorescence labelling. Note that the fluorescent staining widely used for bio-distribution studies of NPs [25,26] suffers from toxicity and photo-bleaching of these labels [38].

In the present paper, we report multi-modal imaging of SiNPs in cancer cells by using methods of linear (HR-SIM and Raman scattering) and non-linear (CARS and TPEF) optics in vitro.

## 2. Results

**Figure 1:**
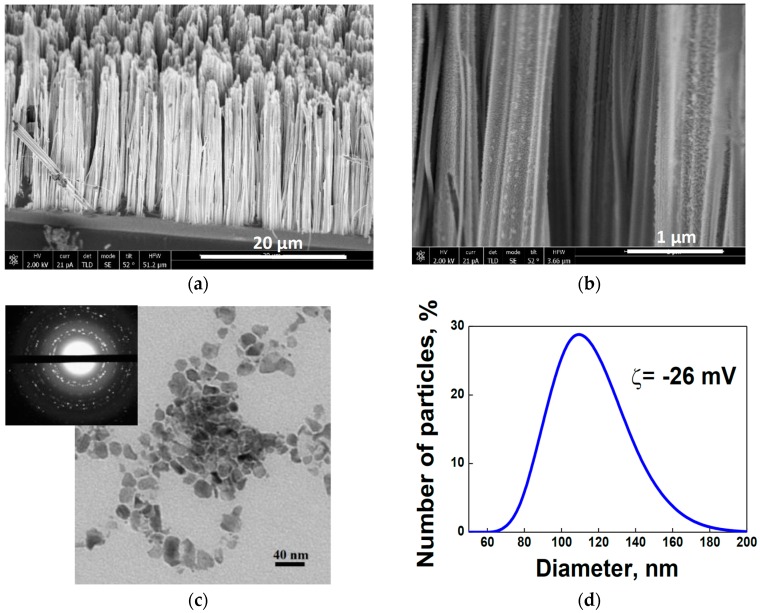

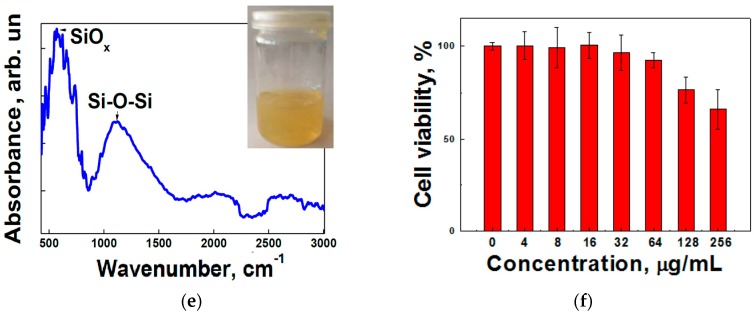
(**a**) Cross-sectional scanning electron microscope (SEM) image of a silicon-nanowire (SiNW) array on crystalline silicon (c-Si) substrate; (**b**) cross-sectional SEM image of SiNWs; (**c**) transmission electron microscopy (TEM) image of silicon nanoparticles (SiNPs) and inset with electron diffraction pattern of SiNPs; (**d**) Dynamic light scattering (DLS) size distribution function of SiNPs; (**e**) Fourier-transform infrared (FTIR) spectrum of SiNPs and inset with digital image of an aqueous suspension with SiNP concentration of 0.25 mg/mL; and (**f**) viability of MCF-7 breast cancer cells vs. SiNP concentration.

Scanning electron microscopy (SEM) shows that the prepared SiNW arrays consist of nanowires with diameters of about 100 nm (see Figure 1a,b). Transmission electron microscopy (TEM) of SiNPs obtained by ultrasonic fragmentation of SiNWs [15] reveals both separated NPs with sizes of 10–40 nm and their agglomerates (see Figure 1c). The inset of Figure 1c shows the corresponding electron diffraction pattern of the SiNPs obtained in “transmission” geometry. The latter consists of bright points located on diffusive rings, which indicate disoriented nanocrystals of the SiNPs.

Dynamic light scattering (DLS) data, exhibited in Figure 1d, show that the mean hydrodynamic diameter of SiNPs in water is about 100 nm and that the zeta potential (ζ) is −25.6 mV. A Fourier-transform infrared (FTIR) spectrum of the dried suspension (see Figure 1e) demonstrates the predominant covering of SiNPs by silicon oxide that is evidenced by the absorption peaks of SiO*_x_* (1 < *x* < 2) and Si–O–Si vibration lines at 508 cm^–1^ and 1070 cm^–1^, respectively. These signals belong to the stretching and symmetric/antisymmetric vibrational modes of the Si–O–Si bridges [69,70]. The oxidation of the SiNP surface is probably due to the last stage of the metal-assisted chemical etching (MACE) process, i.e., nitric-acid treatment [15]. The oxygen coverage of the SiNP-surface is caused by the hydrophilic property of silicon, which together with the negative ζ value determines and proves the stability of the NP suspension (see inset of Figure 1e).

Figure 1f shows results of an in vitro investigation of the cytotoxicity of SiNPs injected into living MCF-7 breast cancer cells. The viability of MCF-7 cells incubated with SiNPs occurs for the NP concentration of 4–128 µg/mL. A decrease to 60% viability is detected for NP concentration above 250 µg/mL. This can be explained by a slowdown of the proliferation rate of the cells [10].

**Figure 2:**
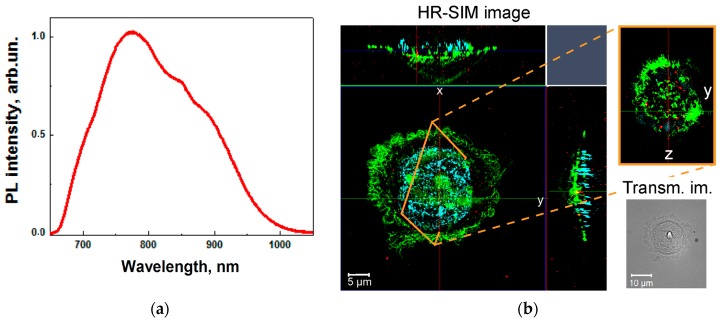
(**a**) Photoluminescence (PL) spectrum of an aqueous suspension of SiNPs; and (**b**) fluorescent HR-SIM image and transmission light image (Transm. im.) of MCF-7 breast cancer cells incubated with SiNPs for 24 h. The cell nuclei were stained with Hoechst 34580 and the cytoplasm actin was stained with Alexa Fluor^®^ 488 Phalloidin (coloured in cyan and green, respectively). The SiNPs are marked in red.

SiNPs in aqueous suspension exhibit PL emission with a quantum yield of about 0.1% and a spectral maximum at 780 nm (see Figure 2a). This PL band can be explained by radiative recombination of excitons, confined in silicon nanocrystals with sizes of 3–5 nm [14]. Such small nanocrystals are supposed to be formed at SiNW sidewalls during the MACE process [71]. As mentioned above, the PL property of SiNPs can be effectively applied for HR-SIM imaging of MCF-7 breast cancer cells transfected with SiNPs. Figure 2b demonstrates that SiNPs (bright red spots in the image) after 24 h of incubation time are efficiently taken up by the cells. In order to obtain a higher contrast during the experiments, cell nucleus and cytoplasm were stained and marked in cyan and green colour, respectively. As can be seen, SiNPs penetrate effectively into the cytoplasm and are localised on the periphery of the nucleus after an incubation time of 24 h. The localisation of SiNPs in the cell cytoplasm was also confirmed by Z-scan imaging (see Figure 2b). It is clearly seen from the images of Figure 2, that during the cell’s mitosis the SiNPs remain in each of the daughter cells.

**Figure 3:**
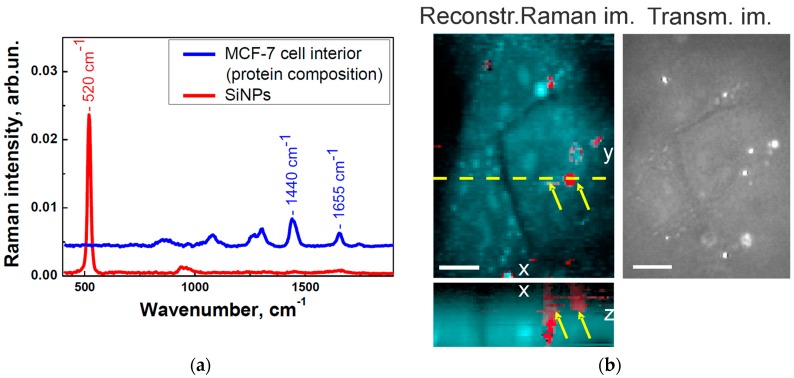
(**a**) Raman spectra of the protein composition (blue line) of the MCF-7 cell interior and of the SiNPs (red line), respectively, after an incubation time of 24 h extracted by applying the vertex-component-analysis (VCA) algorithm; and (**b**) Raman spectroscopy images (xy- and xz-cross-sections of the Raman image reconstructed with VCA) and transmitted-light image (Transm. im.) of MCF-7 cells incubated with SiNPs for 24 h. The SiNPs depicted in red and pointed with yellow arrows are located in the cell cytoplasm depicted in cyan. The scale bar corresponds to 10 μm.

Figure 3 demonstrates a typical Raman image of MCF-7 cells after 24 h of incubation with SiNPs reconstructed by a vertex-component-analysis (VCA) algorithm, as described in Section 4. By performing this analysis, SiNPs can be distinguished from the cell interior. It is known, that the cell interior is emitting the Raman bands of the protein-backbone vibrations, while SiNPs show the characteristic Raman peak at 520 cm^−1^ [17,72,73,74]. Thus, by picking up the spectrum of interest from the set of clusters, two clusters, corresponding to the SiNPs (depicted in red) and to the protein composition of the cells (depicted in blue), have been detected (see Figure 3a). The Raman images of Figure 3b were coloured in accordance with these spectra, which allows us to identify accurately the localisation of SiNPs within the cells. Aiming to ascertain the presence of SiNPs inside the cells rather than on the cell membranes, an additional Z-scan at the defined location (see X–Z cross-section of the cell cluster in Figure 2) was performed.

**Figure 4:**
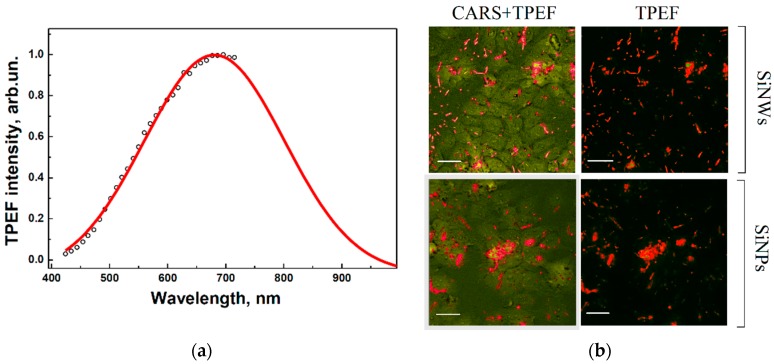
(**a**) Two-photon excited fluorescence (TPEF) spectrum of SiNPs; and (**b**) merged images of CARS and TPEF of SiNWs (**top**) and of SiNPs (**bottom**) in MCF-7 cells. SiNPs depicted in red within the cells depicted in green. The scale bar corresponds to 20 µm.

TPEF and CARS studies were performed by using milled and non-milled SiNWs. Figure 4a shows a typical TPEF spectrum of SiNPs excited by femtosecond laser pulses with wavelength of 750 nm. Basically, this is similar to the one-photon excited PL presented in Figure 2a. Figure 4b (top left) shows merged images recorded by the CARS signal of Si-lattice phonons and the TPEF signal obtained from an image of SiNWs distributed within MCF-7 cells, which was generated with a pump beam at 718 nm. The dominant signal of individual SiNWs, depicted as bright short rods in Figure 4 and obtained by applying both non-linear spectroscopic techniques, can be clearly seen. The TPEF image shown in Figure 4b (top right) confirms the presence of nanowires. While the TPEF regime does not show cellular structure, its application together with the CARS modality allows visualising the distribution of SiNWs within the cells. Similarly to the bioimaging of SiNWs, the simultaneous application TPEF and CARS is an efficient approach to visualise SiNPs inside cells, as shown in Figure 4b, (bottom). Individual nanoparticles as well as their agglomerates are visible as bright spots in the cells.

## 3. Discussion

The obtained results on the linear optical diagnostics of cells with SiNPs applying HR-SIM reveal the possibility to obtain 3D images with extremely high (sub-micrometer) spatial resolution. The necessary condition for HR-SIM measurements is an efficient PL of the small SiNP nanocrystals in the red and near infrared region of the spectrum caused by photoexitation with shorter wavelength, e.g., with blue or UV wavelengths. This requirement is hardly achieved for bioimaging of living cells and deep penetration of biological tissues. This limitation can be overcome by using spontaneous micro-Raman spectroscopy applying excitation with infrared light. Furthermore, the Raman bioimaging can be performed also with non-luminescent SiNPs. An additional advantage of the hyperspectral Raman imaging is the possibility to obtain information about the chemical composition of cells and about the amount of SiNP uptake. Because of the low efficiency of spontaneous Raman scattering the typical acquisition time for bioimaging can cause a measuring time of many minutes and even hours, a duration which is too long and, therefore, hardly applicable for the investigation of living cells. However, the interplay between the hyperspectral Raman and the single-band CARS imaging provides monitoring of the spectral changes of cells and nanoparticles and can be very informative for studies of biodegradation of SiNPs over time.

Thus, bioimaging with simultaneous measurements of CARS and TPEF, as shown here, is able to provide a significant complement to the spontaneous Raman micro-spectroscopy. The presented non-linear optical methods are more attractive due to their higher sensitivity and selectivity as well as their faster acquisition time, which is particularly promising for imaging of living cells. The TPEF signals of SiNWs and SiNPs in the visible and near-infrared ranges were detected by excitation with wavelengths close to the transparency region of biological tissues. Therefore, it can be applied for the diagnostics of thick tissue samples both ex vivo and in vivo. The combination of TPEF and CARS measurements provides additional information about both, the cell morphology and the localisation of SiNPs within cells. In perspective, a time series of CARS images can also be performed additionally in order to monitor the kinetics of SiNP uptake. That can be done more easily by CARS measurements rather than by conventional Raman spectroscopy. Thus both the linear and non-linear optical methods provide important information about different stages of nanoparticle behaviour in living cells that can be crucial for their application in cancer theranostics. Furthermore, these methods can be combined with autofluorescence micro-spectroscopy that is a very efficient approach for in vivo investigation of various intracellular dynamic processes and for three-dimensional characterisation of tissues without administration of any contrast agent.

## 4. Materials and Methods 

### 4.1. Nanoparticle Formation

Aqueous suspensions of SiNPs were prepared by 3 h ultrasonic grinding (37 kHz, 90 W) of SiNWs. Afterwards, the suspensions were centrifuged for 3 min at 2000 rpm and the resulting supernatant was used for the experiments. SiNWs were prepared by metal-assisted chemical etching (MACE) of low boron-doped single-crystalline wafers (doping level: 10^16^ cm^−3^; conductivity: 1–5 Ω·cm) (100). Prior to the MACE procedure the Si substrates were rinsed in 5% HF aqua solution for 1 min to remove native oxide. Then, during the first step of the MACE process, thin (~100 nm) layers of Ag nanoparticles of different morphology were deposited on the substrates by immersing them in aqueous solution of 0.02 M of silver nitrate (AgNO_3_) and 5 M of HF with a volume ratio of 1:1 for 30 s. In the second step, the Si substrates covered with Ag nanoparticles were immersed for 20 min in a solution containing 5 M of HF and 30% H_2_O_2_ with a volume ratio of 10:1, which was filled in a teflon vessel. The etching was performed at room temperature. Then SiNW arrays were rinsed several times in deionised water and additionally immersed in concentrated (65%) nitric acid (HNO_3_) for 15 min to remove residual Ag nanoparticles from the SiNWs. Finally, the samples were rinsed several times in deionised water and dried at room temperature.

### 4.2. Analysis of SiNPs

Structural investigations of SiNW-based samples were carried out by using a field emission scanning electron microscope (FE-SEM, Carl Zeiss ULTRA 55, Carl Zeiss, Jena, Germany) and a transmission electron microscope (TEM, LEO912 AB OMEGA, Carl Zeiss, Jena, Germany). A Malvern Zetasizer Nano ZS instrument (Malvern Instruments Ltd., Malvern, England, UK) was used to determine the size and zeta potential (ζ) of SiNPs obtained from data of dynamic light scattering (DLS). The surface composition of nanoparticles was studied by using a Fourier-transform infrared (FTIR) spectrometer Bruker IFS 66v/S, (Bruker, Karlsruhe, Germany) with a germanium prism for attenuated total reflection. Before recording FTIR spectra, the suspensions were dried in air on the prism surface. The FTIR measurements were done at room temperature in vacuum at a residual pressure of 10^−3^ Torr.

Additionally, the suspensions of SiNPs were characterised by using PL spectroscopy by excitation with an Ar-ion laser (wavelength 364 nm, power 10 mW, spot diameter 1 mm, Spectra-Physics, Stahnsdorf, Germany). The PL signal was detected using a grating monochromator (MS750, SOLAR TII, Moscow, Russia) equipped with a CCD array. The measurements were carried out at room temperature in air.

### 4.3. Cell Cultivation and Sample Preparation

In vitro cytotoxicity experiments were performed with MCF-7 breast cancer cell line. The cells were cultured in culture flask of 25 cm^2^ in DMEM/F12 supplemented with 80 mg/L gentamicin, 20 mМ Hepes, 10% FBS Gibco^®^, and incubated at 37 °C with 5% CO_2_. Then, the cells were seeded into 12 well plate at 1 mL per well at a density of 5 × 10^4^ cells/mL. After 24 h, the cell cultural medium was replaced by cultural medium, which contained SiNPs at different concentrations and incubated for 24 h. The reference cell group was incubated without nanoparticles. The wells then were washed twice with Hank’s saline, and the cells were removed from the surface of the wells by trypsinisation. Whereupon, 1 mL of Hanks’ solution supplemented with 10% FBS Gibco^®^, containing 5 mM of 10(6)-Carboxyfluorescein diacetate *N*-succinimidyl ester (CFSE), (Sigma) and 50 µg/mL Propidium iodide (Biotium) were added to each well and then maintained for 15 min at 37 °C. The number of cells in each well was determined with a cytofluorimeter of Partec PAS III applying an excitation wavelength of 488 nm. The living and dead cells were considered as cells fluorescence signal from that recorded in the FL-1 (517 nm), or FL-3 (617 nm) channel, respectively. The results were statistically processed applying Student’s *t*-test with certainty of 0.95.

For in vitro imaging experiments, a human MCF-7 breast-cancer cell line was used. The cells were cultivated in cell-culture flasks (658175, Greiner Bio-One GmbH, Frickenhausen, Germany) in liquid medium (RPMI 1640, Biochrom AG, Berlin, Germany) with 10% of FBS (Biochrom AG, Berlin, Germany) in the incubator (5% CO_2_ at 37 °C). The Trypsin solution (BioWhiftaker, Lonza, Verviers, Belgium) was used to detach the cells that were later transferred onto CaF_2_ crystal slides. Instead of regular glass slides, CaF_2_ slides were used in further optical measurements in order to avoid interfering background signals. The cells deposited on CaF_2_ slides were cultivated in Petri dishes until approximately 50% confluence was reached. For investigating incubation and interaction processes of MCF-7 cells with SiNPs, slightly boron-doped SiNPs with a final concentration of 50 µg/mL were used.

After 24 h the incubation was stopped by fixation of the cells in 10% formaldehyde (AppliChem GmbH, Darmstadt, Germany), followed by several washing steps in Phosphate Buffered Saline (PBS Dulbecco, Biochrom AG, Berlin, Germany). The CaF_2_ slides were placed in Petri dishes, which were filled with distilled water and stored at +4 °C until the experiments were performed.

### 4.4. High-Resolution Structured Illumination Microscopy

HR-SIM experiments were performed using an optical microscopic system ELYRA-S.1 (Carl-Zeiss, Jena, Germany). With this system, a twofold lateral resolution enhancement in comparison with the conventional wide-field microscope can be achieved by a periodic light pattern, which is realised by inserting a sinusoidal grating in the illumination pathway. It modulates the high-frequency signals from the sample down to frequencies readable by the microscope [75]. The experiments were carried out by illumination at 405 nm, 488 nm and 642 nm excitation wavelengths using specific gratings and filters for each laser line. An oil-immersion objective 63×/NA 1.40 (Carl-Zeiss, Plan-Apo, Jena, Germany) was used for imaging. The images were recorded with an EMCCD array (iXon 885 from Andor, Belfast, UK) cooled at −63 °C and afterwards reconstructed using the ZEN 2010 Carl-Zeiss software (Carl-Zeiss, Jena, Germany).

### 4.5. Micro-Raman Spectroscopy

For the Raman imaging, a confocal Raman microscope (WITec, Ulm, Germany) with an excitation wavelength of *λ*_ex_ = 785 nm and 50 mW laser power was utilised. The glass sample of homogenous monolayer of MCF-7 cells transfected with SiNPs and immersed in Petri dishes filled with distilled water were radiated through a 60×/NA 1.0 water immersion objective (Nikon NIR Apo, Tokyo, Japan). The laser radiation was transmitted by a single-mode optical fiber to the microscope and focused onto the sample. The acquired Raman spectra were recorded by a CCD camera operating at −65 °C. The water surrounding the CaF_2_ slides prevents the cells from thermal destruction during measurements due to the applied high laser power and the long acquisition time. Raman spectra were determined at wave numbers between 400 and 3100 cm^−1^ with an integration time of 0.5 s. The pre-calibration of the system was performed on a single-crystalline silicon slide with a Raman signal of 521 cm^−1^.

For reconstruction of the obtained Raman spectra of SiNP-compounds and clusters distributed within the cancer cells a vertex component analysis (VCA) was applied [18,19,76,77]. By using this algorithm, distinguishing of mostly dissimilar spectra from all measured spectral sets became possible and facilitated to separate these spectra into several clusters of SiNP-compounds. The required components can then be clearly distinguished by extracting particular cluster of interest from the total set of all clusters. An additional data processing (i.e., removal of cosmic rays, baseline correction, control of intensity thresholds and vector normalisation) was performed for all spectra [20,78,79,80,81].

### 4.6. Two-Photon Excited Fluorescence and Coherent Anti-Stokes Raman Scattering

Two-photon excited fluorescence (TPEF) spectra of aqueous suspensions of SiNPs were measured by using a Carl Zeiss LSM-710-NLO confocal microscope (Carl Zeiss, Jena, Germany) equipped with a pulse femtosecond Chameleon Ultra II laser system (Coherent Inc., Bloomfield, NJ, USA), tunable in the 690–1060 nm range. The luminescence spectra of SiNPs was registered by the 32 channel GaAsP detector in the spectral range of 425–725 nm under excitation by a 458, 488, 514, 561, 633 nm CW laser or 730–850 nm 80 MHz pulse laser, with a pulse width of 140 fs. The spectra were extrapolated using Gaussian curves to the long-wavelength region across the border of detectable region.

To visualise SiNPs in tumor cells, the CARS- and TPEF-microscopy approaches were applied [82]. Briefly, the CARS setup consisted of the short-pulse laser source of a Coherent Miro HP Ti sapphire laser pumped by a Verdi-V18 Nd vanadate laser (Coherent, Santa Clara, CA, USA) at 532 nm. The Ti: sapphire laser produced 1 ps-pulses with a repetition rate of 76 MHz, an average power of 3.5 W and wavelengths in the range of 700–1000 nm. A 50:50 beam splitter was used to split the laser beam into two parts to pump two optical parametric oscillators (OPOs) (APE, Berlin, Germany). The OPO allowed tuning the laser wavelength continuously from the visible to the near-infrared spectral range (500–1600 nm, spectral width of the pulses 0.5 nm) with a typical power of 10–20 mW on the sample. The Raman mode at 520 cm^−1^ was used to record CARS images of the cells containing SiNPs. Two OPO beams with wavelengths of 718 nm and 746 nm, respectively, were used for the experiments. A motorised delay stage in combination with a retroreflector and a dichroic mirror was used to recombine both beams and to overlap them in space and time. The beams were sent into a laser scanning microscope LSM 510 Meta (Zeiss, Jena, Germany). The laser radiation was focused by a 20/0.4 objective. The CARS- and TPEF-signals were collected in forward and backward (epi) directions, correspondingly, and were detected by photomultipliers (Hamamatsu R6357, Hamamatsu Photonics Deutschland GmbH, Herrsching am Ammersee, Germany).

## 5. Conclusions

The combination of linear and nonlinear optical techniques for the visualisation of SiNPs in cancer cells was demonstrated for the first time. The HR-SIM and the spontaneous micro-Raman spectroscopy provide the ability to visualise SiNPs and to distinguish them from cell organelles, as well as to accurately identify chemical compounds inside cancer cells. The CARS- and TPEF-microscopies have a high potential for the visualisation of SiNPs, allowing fast imaging acquisition with NIR radiation. This leads during the imaging process to a sufficient increase of penetration depth and scanning region, correspondingly, allowing distinguishing tumour areas from the non-cancerous ones. By these imaging techniques, a study of cancer-tissue morphology with submicron resolution as well as the identification of tumour-cell compositions with high specificity becomes possible. The obtained results reveal new prospects of multi-modal visualisation and precise detection of the uptake of SiNPs by cancer cells, which appears to be very valuable for future widespread theranostic applications.
